# State Boards Versus Incompetent Dental Colleges

**Published:** 1912-02-15

**Authors:** Geo. W. Bowling

**Affiliations:** Member of Oklahoma Board of Dental Examiners, Lindsay, Okla.


### STATE BOARDS VERSUS INCOMPETENT DENTAL COLLEGES.

BY DR. GEO. W. BOWLING, MEMBER OF OKLAHOMA BOARD OF DENTAL EXAMINERS, LINDSAY, OKLA.

A corporation or a society operating a college of dentistry should only be allowed to work under a charter from the U. S. Government, and then be connected with and governed by a high grade university of general and special learning. We are actuated in making this statement by the fact that there is so much inducement for graft connected with the privately owned colleges, and there are so many of them, that even well intentioned men find themselves in such embarrassing situations when financially associated with these schools that they are forced to do things they never would consent to do as private practitioners.

Our young men enter these schools honest intentioned, inexperienced and ready to put forth all their efforts and all the money they can raise, in order to learn the mysteries of this healing art, and be able to minister to the suffering and to give the public good, honest and modern service.

Instead of being carefully and thoroughly prepared in well equipped and honorably conducted schools our graduates of to-day are carried over the many subjects of the curriculum so hurriedly, that they only have a vague idea and little knowledge of the art.

The business methods of a great many of these cheap and inferior schools, have simply implanted in the crudely developed minds of these inexperienced youngsters, methods of dealing that are unfair and dishonest and a grave menace to an innocent public. For instance a great many of these schools, advertise for a clinic, stating that patients will only be charged a minimum cost for material used in the work they may have done. By the end of the junior year the students have learned one thing most vividly if nothing more and that is, that their college makes a substantial profit off their work each month.

Most colleges of this type work their clinics daily and put forth the greatest effort to keep each senior student busy in the clinic every afternoon and most mornings, working for the pocketbooks of the promoters of the college. A great deal of time there is only one demonstrator to every fifteen to thirty-five operators. From four to seven lectures are delivered each day, which is simply worse than "stuffing." The method of having each student turn in each year a certain amount of prosthetic and crown and bridge technic work,—the students purchasing all the materials at local supply houses, and giving it to the college, besides paying a fee annually is an abomination. The deans of most of these colleges must have secured their training selling silver and gold mine stocks or in wild cat real estate agencies. Of course these College Managers want their schools left alone by the law, and neither do they want them connected with the universities for a great many reasons, better known of course to these unworthy teachers of our healing art.

There are instructors in these schools who will affirm that a dental student does not need so much theory, because, they are incapable of teaching it or havn't proper facilities. A man who will make such an unfounded statement needs to be educated himself and is the worst enemy to the profession of dentistry. I would say to those who are now detained in such institutions that the best thing for their future is to get away from such incompetent instruction as quickly as possible, for the betterment of the whole profession.

The time is not far distant, we hope, when every student of dentistry should first have a medical education before he enters into the specialized branch of the healing art, called dentistry. If dentistry is a science why not study it from the scientific standpoint. Art itself is one of the subtlest and most exacting sciences.

If a young man intends entering the profession of dentistry, why does he not enter a university school where in in all probability the teachers employed are among the best of their kind to be had. A professional man loves to look back over his years of experience and attribute at least a portion of his success, in starting at least, was due to the skilled teachers in the institution from which he graduated. And why not have ones degree from one that is known beyond its own door, not as a school of dentistry only, but as a university of learning. Those schools that merely carry a university name, and are not wholly connected with and governed by the university, are poorer schools than those that live on their own name as schools of dentistry only.

The idea prevailing among members of some Examining Boards is blind in that it concludes if a man is a graduate he is all right and must pass their board. I have by actual experience had the matter so vividly impressed on me that I can not believe it for the best interests of the profession or the good of education either.

If the candidate is a graduate, the next question should be from what school does he come. "We examiners have a fairly good record of all the schools. If he is from a school where dentistry is really *taught*, he is all right and will make a good grade, but if he is from a school where dentistry is commercially taught or by the plumbers or carpenters methods the chances are that on theoretic questions he will often make the statement that he "never heard of that question before," or "it is an unfair question." I believe in conducting our examinations to determine if the candidates are educated as professional men, and not as tradesmen.

Here is a point I want to make and it is this—schools that do not maintain an ample standard of curriculum are preparing men as tradesmen and not for a profession. Dentistry should not be taught along commercial lines. The Government should restrain these "wild cat" individuals and call them to halt and demand that all such schools now in operation shall secure a charter from the government. A full report at the end of each year, sworn to by executive officials operating such schools, of every act, teaching facilities on hand, number of students enrolled, number of graduates, amount of money spent in operating clinic.

It seems that something must be done or the profession of dentistry will soon be reduced to a trade. I do not believe that dentistry is practiced on as high a plane of advancement as it was ten years ago. The chief drawback is these inferior schools. I would much prefer a good honest preceptor to any and all such commercial schools. The public demands fair and honest dealing and they are likely to protest a dentist anyway who is simply a dealer and not a minister. It behooves all young men, to certainly make the minutest preparations before going out among a field full of critics to practice such a scientific art as that of dental surgery. It makes little difference whether you or I believe it or not but it always comes to this, no matter what profession one enters—"There will be a survival of the fittest."

I am not persecuting the dental colleges, but I am willing to prosecute every member of the respective faculties who is not fulfilling the function of his position. We should discontinue these inferior colleges and send our students to a university of broad learning to take the dental course.

